# Living on the Edge: Ecological and Evolutionary Dynamics Along Invasion Fronts

**DOI:** 10.1111/ele.70465

**Published:** 2026-10-15

**Authors:** Phillip J. Haubrock, Neil Angelo Abreo, Stelios Katsanevakis, Franz Essl, Ali Serhan Tarkan, Janeide Padilha, Philip E. Hulme, Danish A. Ahmed, António Barbosa Nogueira, Teun Everts, Cang Hui, Ronaldo Sousa, J. Robert Britton

**Affiliations:** ^1^ Department of Life and Environmental Sciences Bournemouth University Poole Dorset UK; ^2^ Faculty of Fisheries and Protection of Waters South Bohemian Research Centre of Aquaculture and Biodiversity of Hydrocenoses, University of South Bohemia in České Budějovice Vodňany Czech Republic; ^3^ College of Health Sciences Mapua Malayan Colleges Mindanao Davao Philippines; ^4^ Department of Marine Sciences University of the Aegean Mytilene Greece; ^5^ Division of BioInvasions, Macroecology and Global Change University of Vienna Vienna Austria; ^6^ Department of Ecology and Vertebrate Zoology, Faculty of Biology and Environmental Protection University of Lodz Lodz Poland; ^7^ Department of Basic Sciences, Faculty of Fisheries Muğla Sıtkı Koçman University Muğla Türkiye; ^8^ CBMA—Centre for Molecular and Environmental Biology/ARNET‐Aquatic Research Network & IB‐S, Institute of Science and Innovation for Bio‐Sustainability, Department of Biology University of Minho Braga Portugal; ^9^ Bioprotection Aotearoa, Department of Pest‐Management and Conservation Lincoln University Christchurch New Zealand; ^10^ Department of Mathematics and Natural Sciences Gulf University for Science and Technology Hawally Kuwait; ^11^ Genetic Diversity Research Institute for Nature and Forest (INBO) Geraardsbergen Belgium; ^12^ Biology Department KU Leuven Heverlee Belgium; ^13^ Department of Mathematical Sciences, Centre for Invasion Biology Stellenbosch University Stellenbosch South Africa

**Keywords:** eco‐evolutionary dynamics, invasive species, range expansion, spatial sorting

## Abstract

Invasion fronts are the advancing edges of non‐native species' ranges and represent dynamic, non‐equilibrium boundaries where colonization, biotic interactions and rapid evolutionary processes interact. Although biological invasions are increasingly well studied, the ecological and evolutionary mechanisms operating at invasion fronts remain conceptually fragmented despite their disproportionate influence on spread dynamics, exerted impact and management. Here, we synthesize how invasion‐front geometries arise from interactions among propagule pressure, landscape permeability, long‐distance dispersal and environmental heterogeneity, producing continuous (including satellite colonies), patchy/fragmented or coalescing fronts that shift with invasion stage and spatiotemporal scales. We integrate ecological and evolutionary evidence from macro‐organisms to show how gradients from core to front can result in declining population density, increased trait divergence, spatial sorting, serial founder effects, expansion load, and behavioural and physiological differentiation. We further draw parallels with climate‐driven range expansions, while emphasizing the stronger disequilibrium and novel biotic contexts characteristic of non‐native invasion fronts. Finally, we map these dynamics onto impact trajectories, demonstrating how trait‐mediated interactions, resource reallocation and system‐level reconfiguration can emerge sequentially along the invasion gradient. By unifying ecological and evolutionary processes across appropriate spatial and temporal scales, we establish invasion fronts as powerful natural laboratories and critical leverage points for predicting, monitoring and managing biological invasions.

## Graphical abstract

Invasion fronts are non‐equilibrium edges where density declines and trait differentiation increases from the core, through a transition zone, to a low‐density, high‐dispersal front. Introductions generate multiple foci and satellite colonies, shaped by environmental filtering, spatial sorting, founder effects, gene flow and expansion load. Depending on context, the front rapidly expands, stalls, or collapses through bottlenecks, maladaptation or Allee effects.

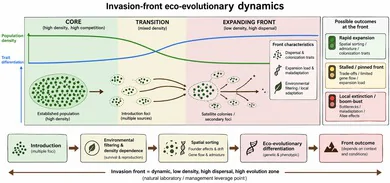

## 1 Why Fronts?

Amid globalization and human driven environmental change, biological invasions are reshaping ecosystems worldwide. Biological invasions describe the human‐mediated introduction, establishment and spread of species beyond their native ranges, where they can cause diverse ecological, socio‐economic and cultural impacts (Haubrock, Everts, Abreo, et al. 2026; Roy et al. 2024). Spatial spread represents the critical gateway between establishment and impact, determining whether a non‐native species' impact remains locally confined or becomes a driver of environmental change (Haubrock, Tarkan, Martín‐Forés, et al. 2026). As such, the capacity for spread is integral to invasion science (Hui and Richardson 2017) and underpins the characterization of a species' ‘invasiveness’ (*sensu* Soto et al. 2024).

### 1.1 Unique in the Extent of Ecological and Evolutionary Dynamics

Considerable work has examined how native and naturalized species evolve and persist at their range limits, where environmental gradients impose strong and relatively static selective pressures and adaptive limits (Radomski 2025; Bufford and Hulme 2023; Angert et al. 2020; Pannell et al. 2019). Previous studies often conform with classical models of geographic range limits that emphasize equilibrium processes that naturally constrain native distributions through interactions among abiotic tolerance, biotic interactions, dispersal limitation and demographic or historical legacies (Radomski 2025; Verzuh et al. 2025; Fagan et al. 2013). More generally, recent work on range edges of native populations has reinforced the view that populations at the front should be treated as dynamic eco‐evolutionary systems rather than static distributional boundaries (e.g., Perrier et al. 2026). Emerging evidence further suggests that such populations may persist in a quasi‐permanent state of non‐equilibrium rather than at a stable abiotic or biotic limit (Lalechère et al. 2025). However, understanding the demographic processes at invasion fronts offers new insights into the eco‐evolutionary processes shaping range margins as they are the boundaries of non‐native species distributions where successful establishment, spread and adaptation intersect (Arim et al. 2006; Phillips et al. 2006). Importantly, the position of the invasion front relative to the species' realized and fundamental niche strongly modulates which processes dominate. Many invasion fronts advance well inside both the species' realized and fundamental niche under strong demographic disequilibrium, where dispersal, propagule pressure (defined as the number of individuals released, the frequency of release events and the quality of the introduced propagules; Lockwood et al. 2005), and spatial sorting tend to predominate over local adaptation. In contrast, many native range edges are more frequently constrained at or near the boundary of the realized niche (Sexton et al. 2009; Angert et al. 2020; Radomski 2025). This distinction helps explain why ecological and evolutionary dynamics at invasion fronts often differ markedly from those at classical range margins. For instance, cane toads ( *Rhinella marina* ) in Australia and round gobies ( *Neogobius melanostomus* ) in North America and Europe show clear core‐to‐front gradients in dispersal‐related traits and behaviour, with individuals at the invasion front exhibiting enhanced mobility and bolder phenotypes compared to individuals from the invasion core (Phillips et al. 2006; Gutowsky and Fox 2012). Similar core‐to‐front gradients in ecologically important traits have also been observed in invasive plants. Garlic mustard ( *Alliaria petiolata* ) populations at the invasion front, for example, allocate greater resources to allelopathic chemical production to suppress naive native competitors, while core populations rapidly evolve reduced chemical investment and place greater emphasis on local competition (Evans et al. 2016). Another example is the genome sizes increasing toward the leading edge in common ragweed ( *Ambrosia artemisiifolia* ), consistent with selection for traits enhancing reproductive success during range expansion (Cang et al. 2024).

Invasion fronts therefore differ from many natural range margins because they usually arise under strong demographic disequilibrium and ongoing human influence, although climate‐driven range shifts in native species can also produce non‐equilibrium edges (Sexton et al. 2009; Angert et al. 2020; Radomski 2025). Invasion fronts are therefore best understood as transient phases of range expansion shaped by novel biotic interactions and feedbacks among dispersal, adaptation, spatial sorting and impact (Phillips et al. 2008; Shine et al. 2011; Urban et al. 2008). Whereas classical treatments of native range limits often emphasize processes that constrain spread at quasi‐equilibrium boundaries, invasion fronts highlight processes that promote and accelerate spread at non‐equilibrium edges. Consequently, invasion fronts are increasingly recognized as transient, heterogeneous, and shaped by interacting ecological and evolutionary processes (Sexton et al. 2009; Angert et al. 2020; Hui and Richardson 2017).

### 1.2 Resulting Invasion Front Typologies

The invasion front is an inherently scale‐dependent feature. Spatially, it can appear as a single wave at broad resolution but often fragments into multiple local *foci* (i.e., discrete, genetically disconnected, spatially separated centres of establishment or spread) at finer scales. An invasion front can take multiple forms, ranging from continuous waves of expansion (including satellite colonies generated by long‐distance dispersal) to patchy/fragmented, or coalescing patterns. These typologies should be interpreted as conceptual phenomena, as similar spatial patterns can arise from different underlying processes and may be difficult to distinguish empirically from occurrence data alone. Importantly, real‐world invasion fronts can represent any combination or intermediate form of these typologies, underlining their interpretation as conceptual phenomena. Invasion fronts may also shift in typology over time as invasions progress through successive stages. Early in an invasion, multiple localized *foci* often emerge from repeated introductions, later coalescing into broader fronts as populations expand and mix (Shigesada and Kawasaki 2002; Kawasaki et al. 2017). Due to endogenous (e.g., local population growth, density dependence, dispersal behaviour and trait variation) and exogenous factors (e.g., habitat heterogeneity, climatic conditions, biotic resistance and anthropogenic barriers), fronts can become heterogeneous (i.e., patchy) or diffuse. Eventually, invasion fronts may form abrupt boundaries where environmental (e.g., niche limits) or biogeographic barriers constrain further spread (Figure 1). An invasion front is thus not just a product of movement, but a consequence of numerous intertwined feedback loops among reproduction, recruitment, habitat permeability and fragmentation (e.g., presence of roads, railroads, dams and canals), biotic interactions and the ability to utilize novel resources (Everts, Deflem, et al. 2025; Balestri et al. 2018). This spatial variability reflects scale‐invariant dynamics that recur from local to continental scales (Liebhold et al. 2020). Short‐ and long‐distance dispersal events from core populations as well as from secondary populations that become additional sources of further spread (i.e., bridgehead effect) combine to shape complex geometries of spread (Everts, Deflem, et al. 2025; Blackburn, Pyšek, et al. 2011, 2015; Mineur et al. 2010). Such patterns have been observed in the American bullfrog ( *Lithobates catesbeianus* ) in Europe, where multiple introduction *foci* and long‐distance dispersal have produced a front characterized by secondary colonies and subsequent coalescent invasion front detectable via environmental DNA (Everts et al. 2021, 2023).

**FIGURE 1:**
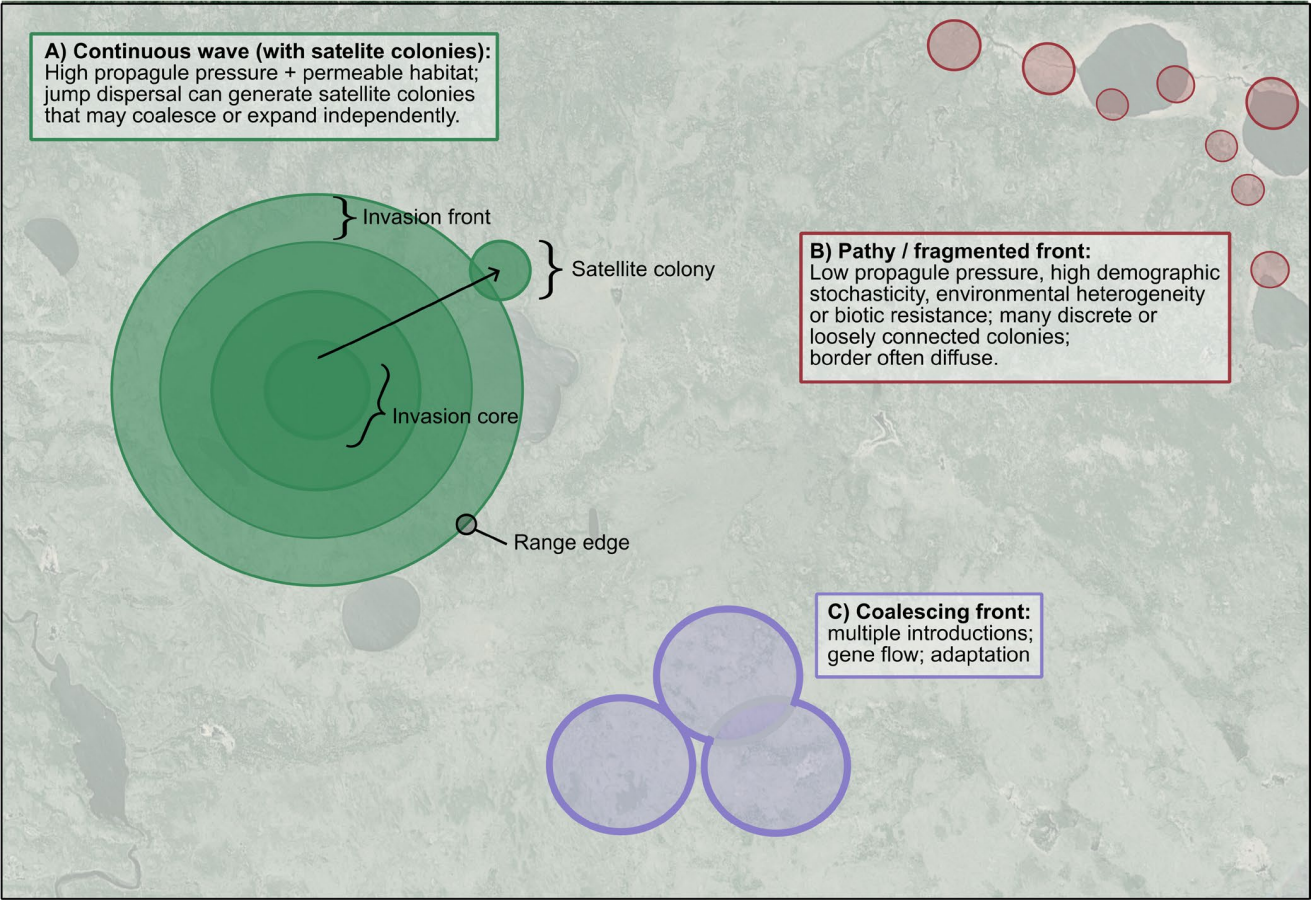
Conceptual illustration of three main typologies of invasion fronts showing how different, potentially overlapping spatial configurations reflect variability in structure and the underlying processes shaping invasion spread across landscapes. (A) Continuous wave (with satellite colonies): A relatively smooth, density‐driven advance under high propagule pressure and permeable habitat; long‐distance jump dispersal can generate satellite colonies ahead of the main front that may expand independently or later coalesce. (B) Patchy/fragmented front: Many discrete or loosely connected colonies of varying size, age and persistence, often producing a diffuse or poorly defined overall border; driven by low propagule pressure, high demographic stochasticity, environmental heterogeneity or biotic resistance. (C) Coalescing front: Multiple independent *foci* (commonly arising from geographically spaced introductions) that expand and merge through gene flow and adaptation, increasing spatial complexity and genetic mixing. Circles represent discrete *foci* or introduction points; shapes are conceptual and not intended to depict actual geographic boundaries. These typologies represent idealized patterns and are not mutually exclusive. Real invasion fronts frequently show intermediate or mixed configurations shaped by multiple interacting processes. This figure is intended as a heuristic tool to illustrate the range of possible invasion‐front structures and their underlying drivers.

### 1.3 Natural Laboratories & Management Opportunities

By observing populations at the invasion front, researchers can witness mechanisms of colonization, adaptation and biotic interactions that often become obscured once an invader is well‐established (Cox 2013; Dominguez Almela et al. 2022; Grayson and Johnson 2018). Even though these mechanisms can also be observed in natural range expansions, the pace and underlying abiotic and biotic processes are fundamentally different in range‐expanding invasions (Radomski 2025). Populations at the invasion front often undergo faster change and differ markedly from populations at the core (the high‐density, demographically stable regions of a population established earlier in the invasion process). As a result, invasion fronts are both natural laboratories for testing ecological and evolutionary theories (Sexton et al. 2009) and strategic control points where management interventions can potentially alter invasion outcomes (Tobin et al. 2011; Sharov et al. 2002). Despite their central role in determining spread and management, invasion fronts remain understudied across ecology, evolution and conservation (Haubrock, Tarkan, Martín‐Forés, et al. 2026). Accordingly, here we highlight why studying invasion fronts matters both conceptually and practically and outline the basis for an integrated framework that embraces the ability of invasion fronts to advance our understanding and management of biological invasions.

## 2 Ecological Drivers at the Invasion Front

Invasion fronts are biologically active zones shaped by interacting ecological and evolutionary processes (Chuang and Peterson 2016). Invasion‐front dynamics can be represented as a progression from continuous waves of population expansion (Skellam 1951; Figure 2A), through patchy or fragmented fronts driven by long‐distance dispersal or additional primary or secondary introductions, followed by the coalescence of multiple introduction *foci* and satellite formation generating complex spatial overlap (Mineur et al. 2010; Figure 2B). These spatial patterns coincide with both ecological and evolutionary gradients (Figure 2C), where population density often decreases but trait differentiation and dispersal capacity increase toward the invasion front (Phillips et al. 2008).

**FIGURE 2:**
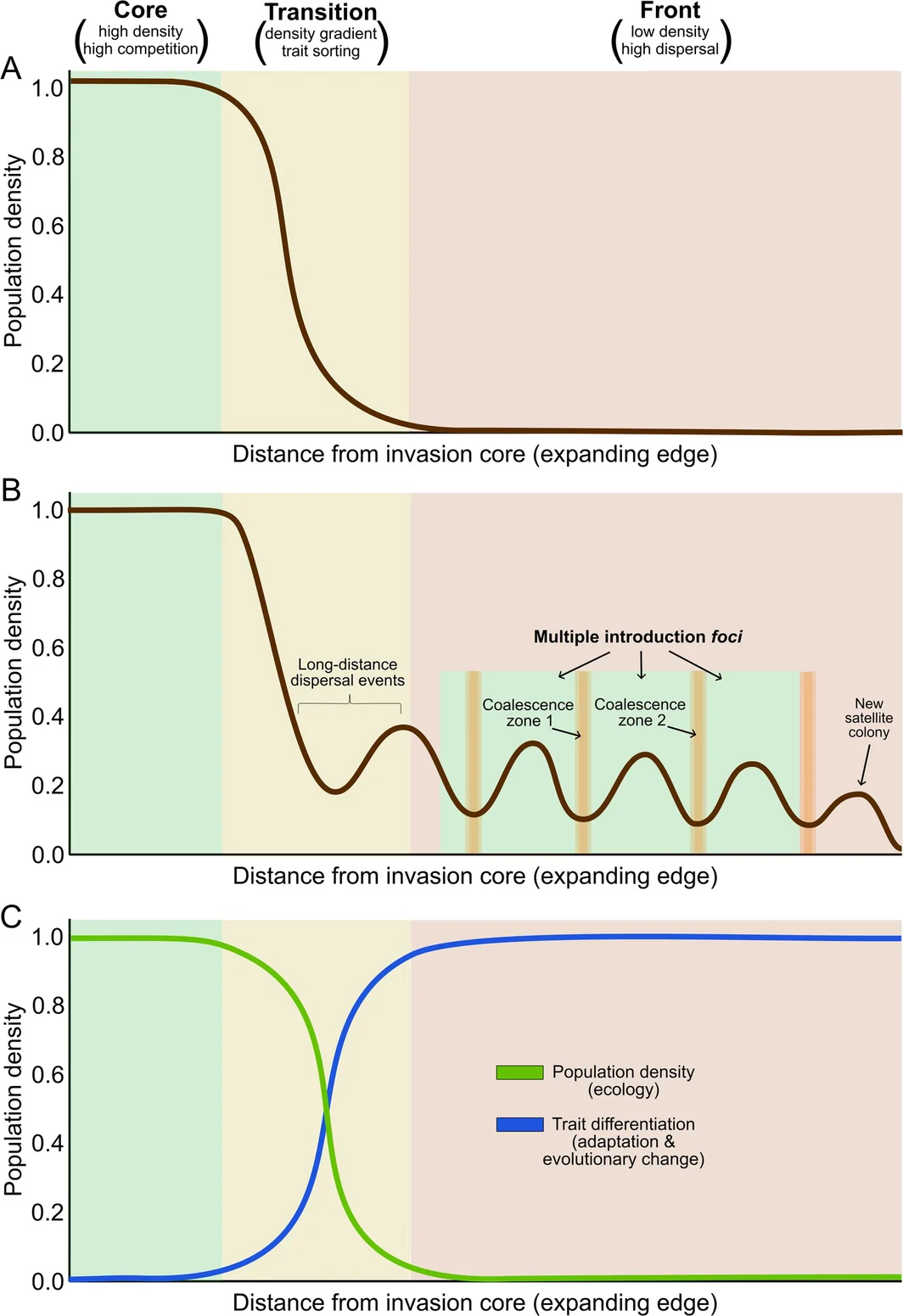
Conceptual representation of some commonly occurring invasion‐front dynamics, shown as the relationship between population density and distance from invasion core, across some exemplary spatial, ecological and evolutionary contexts: (A) A continuous wavefront (a smooth gradient in population density from a high‐density core through a transitional zone of trait sorting to a low‐density, dispersal‐dominated front). (B) A front shaped by long‐distance dispersal and multiple independent *foci*, showing local discontinuities and secondary peaks representing new cores (arising from successful jump dispersal or additional introductions), coalescence zones where expanding fronts from different *foci* meet and mix, and satellite colonies ahead of the main advancing edge. (C) Integrated ecological and evolutionary gradients, in which population density declines while trait differentiation increases toward the front, highlighting the invasion front as a hotspot for ecological dynamics and rapid evolutionary change. Panel (C) illustrates the commonly observed pattern of increasing trait differentiation toward the expanding front driven by spatial sorting and colonization‐related selection; it does not imply that evolutionary processes are absent or weaker in core populations, where selection on other traits may be more efficient due to larger population sizes. The occurrence and detectability of these invasion‐front structures may vary with invasion phase (introduction, lag, expansion or stabilization) and observation scale. Similar spatial configurations can emerge repeatedly at nested scales (i.e., fractal‐like dynamics), reflecting the self‐reinforcing nature of colonization and spread processes (cf. Blackburn, Pyšek, et al. 2011). These scenarios represent idealized conceptual patterns because in empirical systems, multiple processes often interact to generate similar or intermediate configurations, and clear differentiation may not always be possible.

### 2.1 Ecological Drivers of Front Formation

The spatial configurations of invasion fronts are not static, however, and instead evolve concomitantly with the progression of an invasion through distinct temporal phases. Indeed, ecological mechanisms shaping populations at the invasion front can differ between the introduction, early lag phase (i.e., a period of slow population growth and limited spatial spread following an introduction; Crooks 2005), with the duration and character of such lag phases potentially varying among front typologies (due to differences in dispersal, establishment success, habitat connectivity), and demographic stability, acceleration phase of expansion, later stabilization phase and potentially occurring boom‐bust dynamics (Strayer et al. 2017; Blackburn, Pyšek, et al. 2011). Limited propagule pressure and habitat permeability may result in small, fragmented and isolated populations (Briski et al. 2012). Multiple isolated *foci* may arise through repeated introductions or long‐distance dispersal events, forming discontinuous or ‘fragmented’ fronts (possibly mistaken for satellite colonies; Figure 2B). Ecological interactions between these *foci* might initially be weak as establishment remains constrained by geographic distance, demographic stochasticity and sometimes local environmental mismatches. As populations grow and disparate *foci* (i.e., introduced disparate populations or satellite colonies) begin to connect geographically, positive feedbacks between dispersal, reproduction, recruitment, genetic admixture and resource exploitation can emerge. These feedbacks can, in turn, promote front expansion momentum through spatial sorting, admixture and adaptive plasticity, ultimately leading to coalescing or patchy fronts (Figure 2A–C). Eventually, expansions slow and perhaps even halt as populations stop growing in size due to environmental constraints, biotic interactions or density dependence (Urban et al. 2008). Under some conditions, these slowdowns persist, producing ‘pinned’ invasion fronts. In such cases, positive density‐dependent growth and dispersal interact with environmental heterogeneity to halt further expansion, despite apparently suitable habitat beyond the front (Morel‐Journel et al. 2022; Keitt et al. 2001). This range pinning, sometimes described as a form of colonization debt (Morel‐Journel et al. 2023) or niche unfilling (Strubbe et al. 2013), implies that low‐density edge populations can temporarily decouple habitat suitability from realized spread, with important consequences for forecasting invasion trajectories and prioritizing management. The invasion front can thus become diffuse or abrupt depending on habitat heterogeneity, and processes resembling those observed at native range edges (*cf*. Radomski 2025; Angert et al. 2020). At this stage, ecological interactions may intensify and result in either increased population abundance, transforming the former front into a newly established core, while the invasion front continues to advance, or unsuitable environmental or biological conditions that effectively limit additional spread.

These invasion stages, and thus invasion‐front dynamics, can deviate from isotropic diffusion (i.e., equal spread in all directions) and instead show anisotropic spread (i.e., directionally biased spread) imposed by environmental heterogeneity (Hastings et al. 2005). Freshwater ecosystems often generate strongly directional spread through dendritic network topologies and hydrological flow (Altermatt and Fronhofer 2018; Osawa et al. 2013; Leprieur et al. 2008). Marine environments offer broader connectivity but remain structured by persistent currents, retention zones and mesoscale circulation that create patchy and directionally biased spread (Michie et al. 2024; García‐Gómez et al. 2021; Pringle et al. 2011). Terrestrial landscapes can also impose anisotropy through habitat permeability, corridors and barriers such as land use and elevation (Fraser et al. 2015; McRae and Beier 2007). Thus, although biotic and abiotic constraints differ among realms, invasion fronts predominantly reflect spread resembling anisotropic diffusion shaped by landscape structure, dispersal mode, species life history and ecological plasticity (Haubrock et al. 2024; Hastings et al. 2005).

### 2.2 How Spread Becomes Establishment and Impact

At low population densities near the invasion front, demographic feedbacks (e.g., Allee effects) and environmental stochasticity can influence the probability of persistence and the rate of further expansion (Blackburn et al. 2016; Keitt et al. 2001; Sakai et al. 2001), while adaptive plasticity and flexible resource use promote continued advancement (Luo et al. 2019). Concomitantly, niche construction (Odling‐Smee et al. 2003) at the invasion front may play a critical role in determining invasion dynamics and long‐term ecosystem impacts. As populations expand into new habitats, individuals can actively modify local environmental conditions through processes including soil alteration, nutrient cycling, physical engineering, resource depletion and facilitation mechanisms (Ferraro et al. 2025; O'Loughlin and Green 2017). These modifications potentially enhance invader fitness, creating positive feedback that accelerates range expansion. In some cases, these feedbacks involve facilitative interactions with other non‐native species, linking invasion‐front dynamics to the concept of invasional meltdown (Simberloff and Von Holle 1999). For example, the spread of introduced pines can depend on co‐introduced ectomycorrhizal fungi, and other invasions are facilitated by non‐native mutualists or ecosystem engineers that improve establishment conditions (Nuñez et al. 2009). In addition, niche‐constructing traits may interact with spatial selection at invasion fronts to favour phenotypes that both disperse effectively and engineer locally beneficial conditions, potentially leaving lasting evolutionary legacies. The invasion front of a non‐native population thus functions as an ecological interface, often manifesting as pulses, accelerations and decelerations rather than smooth diffusion (Urban et al. 2008).

## 3 Evolutionary Insights at the Invasion Front

### 3.1 Evolutionary Processes Affect the Spread at Invasion Fronts

Across ecosystems, individuals at the invasion front can differ from those in the transitional zone (i.e., the geographically intermediate region between the invasion core and front; Table 1; Kubisch et al. 2014). During an introduction, individuals are subjected to environmental filters and density dependent processes (Figure 3A). Range‐expanding populations encounter and potentially adapt to novel selective environments, and the process of expansion itself can drive evolutionary change through mechanisms such as spatial sorting, genetic drift and serial founder effects (Figure 3B; Van Petegem et al. 2016). Consequently, range expansion often leads to evolved differences in reproductive life‐history and dispersal traits between individuals in the range core and those at the front (Peischl et al. 2013). At the front, individual animals often express enhanced body condition, size, boldness and/or exploratory behaviours (Alves et al. 2025; Damas‐Moreira et al. 2019; Myles‐Gonzalez et al. 2015; Rebrina et al. 2015; Laparie et al. 2013), whereas individual plants at the front may have faster germination and greater capacity to self‐fertilize (Liu et al. 2021; Tabassum and Leishman 2018, 2019). Such front‐associated traits can facilitate dispersal, resource acquisition and competitive dominance. Moreover, low‐density populations at the invasion front often benefit from reduced intraspecific competition and abundant resources, enabling the emergence of density‐dependent traits, such as higher physiological condition and fecundity (Rebrina et al. 2015; Lopez et al. 2012). Yet trade‐offs are common, with selection for dispersal often reducing reproductive investment, as observed in *Rhinella marina* , where morphological evolution increased invasion speed but lowered fecundity in individuals at the front (Hudson et al. 2015, 2016). Similar *r*‐selected strategies are documented in fishes at invasion fronts, where rapid growth, early maturation and high reproductive effort can sustain population expansion (Tarkan et al. 2021; Gutowsky and Fox 2012; Bøhn and Amundsen 2004). These dynamics may be affected by male‐biased dispersal and density‐driven shifts in sex ratio (Chuang and Peterson 2016; Marentette et al. 2011). Conversely, individual animals at the core, where higher population densities generally increase intraspecific competition, may be of lower physiological condition and smaller body sizes, as well as exhibiting less bold, exploratory and aggressive behaviour (Alves et al. 2025; but see Hudina et al. 2015). However, individuals at the invasion front may encounter differing densities of enemies (predators, competitors, parasites, diseases) and mutualists, environmental conditions and resource availability. These differences potentially translate into reduced fitness relative to individuals in core populations, especially when small population sizes at the front allow genetic drift to override selection, enabling deleterious alleles to spread along the invasion front during repeated founder events (Slatkin and Excoffier 2012; Edmonds et al. 2004). Consequently, fecundity at the front may decline relative to the core due to trade‐offs among dispersal, reproduction and competitive ability.

**TABLE 1:** Representative empirical examples of invasion‐front dynamics and ecological and evolutionary factors underlying or driving spread. This table is illustrative rather than comprehensive and highlights representative systems in which factors affecting invasion‐front expansion have been described in detail.

Taxon/system	Region/ecosystem	Factor(s) associated with expansion	Reported invasion‐front pattern or outcome	References
*Rhinella marina* (cane toad)	Australia/terrestrial	Spatial sorting, dispersal evolution, morphological and physiological change	Increased invasion speed and dispersal‐enhancing phenotypes at the front	Phillips et al. 2006; Hudson et al. 2016; Urban et al. 2008
*Neogobius melanostomus* (round goby)	North America, Europe/freshwater	Life‐history variation, sex‐biased movement, behavioural variation	Core–front differences in growth, movement and behaviour during expansion	Gutowsky and Fox 2012; Marentette et al. 2011; Groen et al. 2012; Thorlacius et al. 2015
*Pacifastacus leniusculus* (signal crayfish)	Europe/freshwater	Trait variation, aggression/behaviour, dispersal trade‐offs	Front populations differ in traits and impacts along invasion gradients	Rebrina et al. 2015; Hudina et al. 2015; Alves et al. 2025; Gonçalves et al. 2025; Carvalho et al. 2025
*Lithobates catesbeianus* (American bullfrog)	Europe/freshwater	Multiple source populations, long‐distance dispersal, habitat configuration, detectability via eDNA	Rapid spread shaped by multiple introductions; invasion fronts and hubs identified with eDNA	Everts et al. 2021; Everts et al. 2023; Everts, Deflem, et al. 2025
*Acridotheres tristis* (common myna)	South Africa/terrestrial	Spatial sorting, sex‐biased dispersal, morphological variation	Morphological traits correlate with distance from range core	Berthouly‐Salazar et al. 2012; Berthouly‐Salazar et al. 2013
*Mikania micrantha* (mile‐a‐minute weed/vine, climbing hemp vine, American rope, Chinese creeper)	Asia/terrestrial plant	Rapid evolution of dispersal‐related traits	Dispersal‐related traits shift during range expansion	Huang et al. 2015
*Galinsoga quadriradiata* (haggy soldier, hairy galinsoga, Peruvian daisy or fringed quickweed)	Mountain ranges/terrestrial plant	Dispersal‐related trait shifts along expansion gradient	Trait changes associated with range expansion into novel environments	Liu et al. 2021
*Plantago virginica* (Virginia plantain)	Terrestrial plant invasion	Phenotypic plasticity, rapid adaptation	Plasticity and adaptation contribute to invasion success	Luo et al. 2019
*Pinaceae* spp./conifers with ectomycorrhizae	Argentina and other introduced ranges/terrestrial plant	Mutualist availability, fungal dispersal limitation, belowground facilitation	Spread constrained away from plantations by lack of compatible ectomycorrhizal fungi	Nuñez et al. 2009; Le Roux et al. 2017; Spear et al. 2021
*Neogale vison* (American mink)	Scotland/terrestrial	Landscape heterogeneity, habitat permeability	Spread shaped by heterogeneous landscape structure	Fraser et al. 2015
Marine invaders (multiple taxa)	Coastal/marine	Jump dispersal, secondary introductions	Fronts, jumps and secondary introductions produce distinct spread patterns	Mineur et al. 2010
Riverine fish invasions (multiple taxa)	River networks/freshwater	Network configuration, habitat quality, biological traits	Directional and constrained spread shaped by river topology	Dominguez Almela et al. 2022; Rubenson and Olden 2017

**FIGURE 3:**
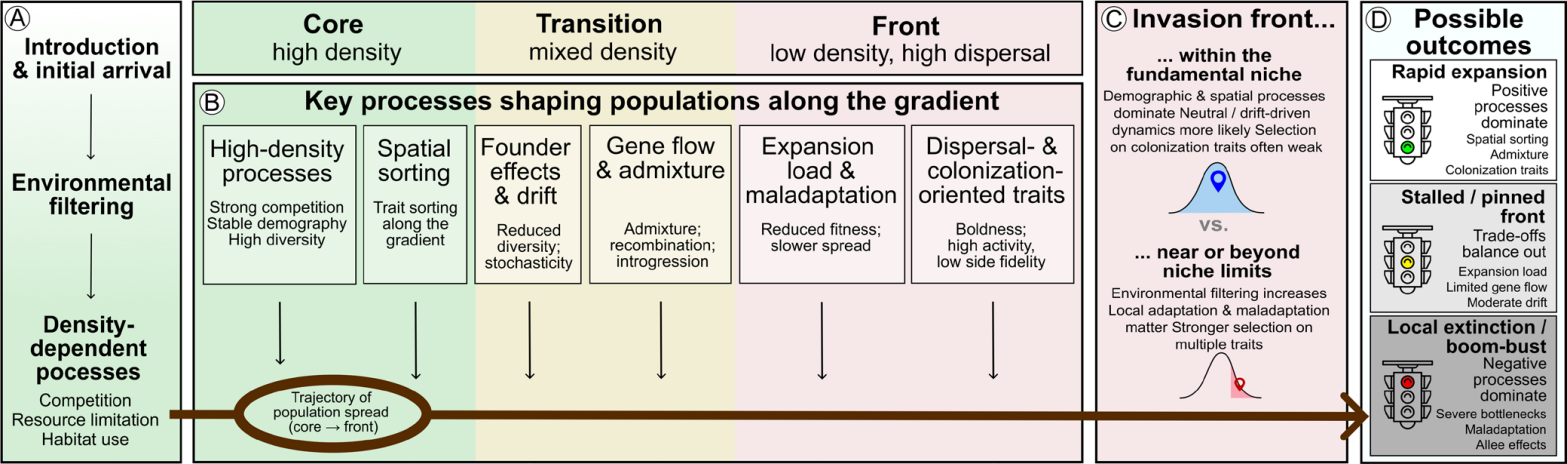
Conceptual synthesis of eco‐evolutionary dynamics along the invasion gradient from core to expanding front. Following an introduction event, populations experience environmental filtering and density‐dependent processes in the core (A). As individuals disperse outward from the core, spatial sorting, serial founder effects and genetic drift increasingly shape populations in the transition and front zones, often favouring dispersal‐ and colonization‐oriented traits (e.g., boldness, high activity, low site fidelity). Simultaneously, expansion load, reduced genetic diversity and maladaptation can accumulate, while gene flow from the core, admixture and recombination can restore variation and facilitate evolutionary change (B). At the core and when the invasion front advances well inside the fundamental niche, demographic and spatial processes typically predominate, whereas near or beyond niche limits, environmental filtering and local adaptation become increasingly important (C). The balance of these interacting processes determines three main possible outcomes at the invasion front (D): (i) rapid expansion, when positive processes such as spatial sorting and admixture dominate; (ii) a stalled or pinned front, when trade‐offs between expansion load, limited gene flow and colonization traits balance out; or (iii) local extinction or boom‐bust dynamics, when negative processes such as severe bottlenecks, maladaptation and Allee effects dominate. Traffic light symbols indicate the three possible outcomes at the expanding edge.

The relative importance of these eco‐evolutionary processes is strongly modulated by the position of the invasion front relative to the species' fundamental niche (Figure 3C). When fronts advance well inside the fundamental niche, neutral demographic processes (e.g., spatial sorting, serial founder effects and expansion load) typically predominate (Miller et al. 2020). In contrast, when fronts approach the margin of the fundamental niche, stronger selective pressures favour local adaptation (Angert et al. 2020). Recurrences of these evolutionary core‐front patterns across ecosystems and taxonomic groups indicate that the processes of introduction and establishment are repeatedly recreated at invasion fronts (Blackburn, Pyšek, et al. 2011), reinforcing invasion momentum through the transition of front populations into new invasion cores. Low population densities, high dispersal and rapid population turnover can, however, promote both spatial sorting and selection for traits that enhance range expansion (Shine et al. 2011; Ochocki and Miller 2017). Evolutionary processes at the invasion front consequently arise from strong spatial and demographic structuring that creates distinctive and selective environments, making invasion fronts high risk, high reward habitats for non‐native species. Moreover, demographic stochasticity at low densities means that even small phenotypic advantages can disproportionately influence front dynamics, allowing traits that confer only marginal dispersal benefits to sweep rapidly along the invasion front. While ecological and evolutionary mechanisms determine where a non‐native species spreads (Figure 3D), it remains unclear which processes determine the speed and consistency of this spread (Melbourne and Hastings 2009; Ochocki and Miller 2017).

### 3.2 Parallels and Contrasts With Native Range Expansions

Similar mechanisms and processes are recorded in introduced non‐native diseases and pathogens spreading among native and non‐native hosts (Hulme et al. 2020; Perrier et al. 2026). This is also the case for native species undergoing range expansions under climate and land‐use change (so‐called neonative species; Essl et al. 2019). These populations often experience similar evolutionary pressures at their leading edges, including low density, strong selection for dispersal and rapid reproduction, and serial founder effects. This parallel indicates that invasion fronts and climate‐driven range margins operate under convergent evolutionary dynamics. Indeed, the potential role of assisted gene flow in enhancing adaptation and evolutionary potential at these leading edges has been highlighted as a key management option under climate change (Aitken and Whitlock 2013). Transplant experiments and elevational range‐limit studies have further revealed latent spread potential under shifting climatic regimes (Bufford and Hulme 2023; Pannell et al. 2019). Although both non‐native and native range expansions are driven by and subject to similar evolutionary principles, invasion fronts differ fundamentally in their boundary conditions and evolutionary context. Unlike native (including neonative) species, non‐native species expand into novel ecological and phylogenetic arenas, frequently encountering competitors, predators and resources with which they share no coevolutionary history (Roy et al. 2011). Consequently, the behavioural and ecological cues guiding movement at invasion fronts may differ profoundly among sites and from those in the native range. Non‐native individuals must interpret unfamiliar environmental gradients and novel biotic interactions, potentially shifting the relative importance of abiotic (e.g., temperature, salinity) and biotic (e.g., predation, competition, facilitation) signals. This absence of historical constraint of non‐native species, coupled with often repeated introductions and strong human mediation, can amplify selection for dispersal, plasticity and rapid adaptation far beyond the typical for range shifts of native species (Stewart et al. 2015). Moreover, the repeated, sometimes constant influx of novel genotypes from multiple introductions typical to many biological invasions can generate admixture at the invasion front, increasing evolutionary potential relative to native range expansions (Roman and Darling 2007). Accordingly, invasion fronts provide highly informative model systems for studying eco‐evolutionary concepts in real time, where demographic disequilibrium and ecological novelty interact to accelerate evolutionary responses.

### 3.3 Behavioural and Genetic Mechanisms at the Invasion Front

Spatial sorting refers to the non‐random accumulation of dispersive individuals at the invasion front and can enhance the prevalence of traits that facilitate movement. The magnitude of spatial sorting can be so profound that it overrules classical fitness trade‐offs, such as *r*‐ versus *K*‐strategists (Shine et al. 2011; Alford et al. 2009). Successive generations at the front may thus become increasingly composed of phenotypes optimized for colonization rather than intraspecific competition. As dispersive individuals mate assortatively and pass on traits that accelerate spread, this process could drive rapid evolutionary changes without the need for strong selection (Courant et al. 2019; Huang et al. 2015; Phillips et al. 2010). For instance, the European foxglove ( *Digitalis purpurea* ), invasive in the Americas, rapidly evolved longer proximal corolla tubes and altered nectar rewards within decades of acquiring hummingbirds as novel pollinators, a shift from its native bumblebee pollination syndrome. This demonstrates how novel biotic interactions in the invaded range can drive rapid co‐evolutionary change and local adaptation in non‐native species (Mackin et al. 2021). In turn, this spatial self‐organization contributes to evolutionary acceleration, as observed in cane toads, where morphological and physiological adaptations (e.g., longer limbs, greater endurance) increased their invasion speed fourfold across eight decades (Hudson et al. 2016). Sexual dimorphism, characterized by female‐biased dispersal, and morphological variation, particularly in female wing and head traits, in the invasive common myna ( *Acridotheres tristis* ) are strongly correlated with distance from the core (Berthouly‐Salazar et al. 2012), suggesting that individuals at invasion fronts often exhibit proactive behavioural syndromes (boldness, exploration, low site fidelity) and thereby promote colonization in some contexts (Wong and Candolin 2015; Chapple et al. 2012; Sih et al. 2012; Holway and Suarez 1999). However, such traits do not necessarily increase fitness, because expansion at invasion fronts depends on dispersal capacity, the fitness cost of dispersal and the strength of genetic drift, which can reduce genetic diversity and constrain adaptation (Polechová 2018). Indeed, this has been shown in non‐native round gobies ( *Neogobius melanostomus* ) and signal crayfish ( *Pacifastacus leniusculus* ) being equally or even less aggressive at invasion fronts (Hudina et al. 2015; Groen et al. 2012; but see Gonçalves et al. 2025). Such ‘proactive’ phenotypes are consistent with the pace‐of‐life syndrome, linking boldness and high activity to elevated metabolism, faster growth and *r*‐selected reproductive strategies (Raffard et al. 2022; Réale et al. 2010; Biro and Stamps 2008). Moreover, these underlying behavioural traits promote invasion momentum by coupling dispersal capacity with rapid resource exploitation, although highly dispersive individuals may exhibit reduced competitiveness or fecundity once densities increase, as observed in fish and crayfish invasions (Tarkan et al. 2021; Hudina et al. 2015; Groen et al. 2012). Together, these patterns signify that invasion fronts often operate under distinct behavioural–evolutionary regimes compared to range cores, with trait combinations that maximize expansion but may incur performance costs as populations mature and densities rise.

Beyond behavioural and life‐history traits, additional genetic processes can further shape evolutionary trajectories at the invasion front. Serial founder effects, genetic drift and demographic bottlenecks can reduce genetic diversity and generate an accumulation of deleterious alleles that can constrain population performance (i.e., ‘expansion load’; Peischl and Excoffier 2016; Peischl et al. 2013; Excoffier et al. 2009; Klopfstein et al. 2006). Conversely, gene flow from the core can restore genetic diversity and improve adaptive potential at the front, although excessive influx can dilute local adaptation and slow expansion (Beer et al. 2024; Berthouly‐Salazar et al. 2013). Hybridization between related ‘lineages’ at the front can create novel genotypes with enhanced invasiveness, as seen in fungal and fish invasions (Hessenauer et al. 2020; Diedericks et al. 2018), illustrating how genetic and behavioural evolution can jointly determine the persistence and speed of spread, sometimes outpacing abiotic constraints (Ochocki and Miller 2017; Szűcs et al. 2017). These interacting genetic processes together create an evolving mosaic of local adaptation, maladaptation and admixture along the expanding range edge, producing spatially variable evolutionary outcomes that feed back into spread dynamics.

## 4 Impacts at the Invasion Front

Each invasion represents a unique spatio‐temporal trajectory shaped by local context, propagule pressure and species‐specific traits, as invasions expand not only in space but also in functional depth (i.e., the breadth and intensity of ecological functions, trait expression and impacts within recipient ecosystems) (Haubrock et al. 2024). Consequently, impact type and magnitude vary both among species and populations of the same species (e.g., as a function of population density) and along the invasion gradient itself (Sousa et al. 2024; Shine 2010). Understanding impact dynamics at the invasion‐front requires viewing impacts as evolving properties of the invasion process rather than fixed outcomes. Indeed, although empirical quantification remains scarce, the mechanistic drivers of such stage‐dependent impacts suggest predictable transitions from acute, transient disturbances to chronic, system‐level ecosystem reconfiguration. Importantly, however, these transitions are not merely temporal but spatially structured and therefore neither static nor uniform, arising from the shifting balance between the invader traits, advancing populations (i.e., the invasion front and following zones) and ecosystem characteristics they invade, including biotic resistance and environmental feedbacks (Shine 2010). Recognizing and measuring these transitions across invasion stages would provide a powerful framework for linking population dynamics with ecosystem impact trajectories, a relationship still largely unexplored in invasion ecology.

Early in the spread process, newly established front populations may trigger short‐term disruptions, such as altered resource flows, behavioural shifts in native species or temporary dominance due to predator or competitor naïveté (Stewart et al. 2021; Anton et al. 2020; Heavener et al. 2014). Behind the front, local adaptation, biotic interactions and density regulation progressively modify community composition and ecosystem function. For instance, by exploiting novel resources and modifying nutrient fluxes, expanding populations may reshape local food webs and biogeochemical cycles, thereby influencing resource subsidies and ecosystem functioning both ahead of and behind the moving front (Alves et al. 2025; Lopes et al. 2025). In some circumstances, populations at the front even exhibit higher concentrations of certain potentially toxic elements, which may be associated with a distinct diet, a higher trophic position, more exploratory behaviour and lower epibiotic associate load (Gonçalves et al. 2025). However, the reverse can also occur, with for examples, plants at the front being naive and having lower levels of defensive secondary metabolites, whereas populations established in the core having evolved more chemicals (Gruntman et al. 2017). Such physiological and trophic shifts indicate that front individuals can occupy different ecological niches than core individuals, implying that impacts propagate not only through abundance but through trait‐mediated pathways (van Kleunen et al. 2010). Concomitantly, population abundances at the front often remain lower than in the core, indicating that total impact at the front may be assumed to be lower (Parker et al. 1999). However, *per capita* impacts may ultimately be higher at the front because individuals face less intraspecific competition and are often larger, more aggressive, bolder and have higher metabolisms (Alves et al. 2025). Impacts may thus shift from direct interactions at the front to indirect, system‐level alterations as populations mature and stabilize, representing a temporal sequence in which both the invader's traits and the invaded system co‐evolve and co‐adapt (Dostál 2024). In such cases, invaded systems may undergo additional changes through ‘relaxation’ phases after the front passes, in which impacts may persist through legacy effects or subside as new equilibria emerge (Cuddington 2011).

## 5 Management at the Invasion Front

### 5.1 Complex Core‐Front Dynamics Cannot Be Managed With Uniform Approaches

Invasion fronts are regarded as strategic management zones where intervention can meaningfully alter invasion trajectories (Hulme 2006). Indeed, management actions targeting biological invasions beyond an early stage are often most effective and economically efficient when implemented at or ahead of the advancing front. Specifically, targeting populations from the front and progressing toward the core is generally most beneficial for preventing reinvasion following management interventions (Epanchin‐Niell and Wilen 2012). However, in practice, management often focuses on core populations, as the number of individuals removed tends to be much higher compared to the front, giving the impression of a higher removal efficiency and thus a higher return on investment. Although managing long‐established core populations may produce localized benefits, such as reducing local impacts (Green and Grosholz 2020) or limiting the production of propagules capable of reaching invasion fronts via long‐distance dispersal events (Everts, Deflem, et al. 2025), such interventions typically only slow, rather than prevent, overall spread (Pepin et al. 2019). Moreover, suppressing core populations can generate unintended ripple effects, facilitating the emergence of new satellite populations, ultimately resulting in multiple secondary fronts (Liebhold and Bascompte 2003; Shigesada and Kawasaki 1997). Thus, management strategies that focus disproportionately on established cores risk overlooking the dynamic processes that sustain spread at the front, where intervention leverage is highest on the scale of the complete invasion. Management should therefore be strategically allocated across invasion fronts, satellite populations and core source populations, as the most effective approach depends on whether the front is continuous or fragmented, the relative contribution of each component to spread, and the available resources (Hulme 2006).

Management is hierarchical and management objectives vary with contexts, stakeholders and perspectives, meaning that some situations warrant the management of core populations. However, differences in the eco‐evolutionary dynamics between core and front populations may require control methods to be tailored specifically to either core or front populations. Because individuals at the invasion front can differ phenotypically and behaviourally from those in the core (Llewelyn et al. 2010), effective management may require trait‐informed and stage‐specific approaches rather than a uniform strategy. For instance, the invasion front of non‐native bullfrogs is pushed forward through juveniles being the dispersive life stage, yet management is often conducted with methods that numerically work best for tadpoles (Everts et al. 2022). In practice, this means that methods may need to be adjusted case by case to account for differences in detectability, dispersal propensity, aggression or capture susceptibility between front and core populations. Regardless of the methods used, populations at the front sometimes exhibit distinct traits, such as greater exploratory behaviour or aggressiveness (Hudina et al. 2015; Thorlacius et al. 2015; Groen et al. 2012), which for some species could increase *per‐capita* capture efficiency at the front versus the core. Because populations at the front are typically small, spatially restricted and demographically unstable, early intervention is more feasible before populations establish (Liebhold and Bascompte 2003; Leung et al. 2002; Sharov et al. 2002). To exploit these management opportunities, a strong understanding of the species' distribution is essential (Everts et al. 2023).

### 5.2 Tools to Support the Management of Invasion Fronts

Accurately delineating and forecasting invasion fronts (on a macro‐ecological scale commonly done with Species Distribution Models; Srivastava et al. 2019) often faces inherent flaws due to non‐equilibrium dynamics of the invasion front, which calls for dynamic species distribution models that consider both the non‐equilibrium nature (Hui 2023) and the transitions of selection forces along the core‐front gradient. Improving the management of invasive species requires emerging monitoring tools with greater sensitivity (Fricker and Olden 2023), such as environmental DNA (Everts et al. 2021), real‐time acoustic, radio or PIT telemetry (Meira et al. 2024; Wood et al. 2024) and remote sensing (Gränzig et al. 2023), which is increasingly supported by citizen science as well as local ecological knowledge (Gervazoni et al. 2023). These tools differ in the signals they capture compared with conventional monitoring tools and, therefore, in their identification of invasion fronts in practice. Environmental DNA can generally detect low‐density populations and isolated foci ahead of the visibly occupied range, making it useful for accurately delineating fragmented fronts and locating satellite populations before coalescence (Everts et al. 2021, 2023; Bylemans et al. 2016). Real‐time acoustic, radio or PIT telemetry monitoring can track front movement in invaders, while remote sensing can map the front extent where invaders generate detectable habitat‐level signatures, such as changes in vegetation structure (Wood et al. 2024; Gränzig et al. 2023). Remote sensing provides the opportunity to integrate historical imagery to trace the spatiotemporal dynamics of an invasion, permitting detailed analysis of demographic processes at the invasion front (Sprague et al. 2021). However, direct demonstrations of fully integrated front detection and front‐specific management remain limited for many taxa, highlighting an important gap between monitoring capability and operational control. Complementary to these approaches is landscape genetics, which analyses the relationship between genetic connectivity and landscape features, and can reveal intra‐population barriers to gene flow that appear to be continuously distributed, thereby revealing cryptic ‘internal invasion fronts’ with important implications by providing windows of opportunity for management (Everts, Deflem, et al. 2025; Sherpa et al. 2020).

### 5.3 The Need for a Tiered Management Approach

The spatial form of a front largely determines management success. Continuous or ‘pushed’ wavefronts are comparatively easy to forecast and control, whereas patchy or fragmented fronts pose a larger management challenge. Fronts are thus composed of multiple asynchronously established foci each with unique front characteristics shaped by local abiotic and biotic conditions (Zhao et al. 2019). Managing such fronts offers multiple important lines for intervention. First, management should focus on preventing the establishment of new *foci* by limiting additional human‐mediated introductions or long‐distance dispersal events, as preventing a new population from getting a foothold is more cost‐efficient than removing it once established (Keller et al. 2007; Leung et al. 2002). This requires a multifaceted and layered approach, consisting of policy interventions, decontamination strategies (e.g., Check, Clean, Dry) and a good understanding of a species' spread dynamics across a given landscape matrix (Anderson et al. 2015). Second, the management of patchy or fragmented invasion fronts should prioritize preventing the coalescence of existing *foci*, as multiple small and spatially isolated populations are considerably easier to contain or eradicate than a single, large, continuous population, mainly due to the proportionally larger coverage of front relative to core areas (Bohling 2016). Moreover, limiting coalescence reduces opportunities for genetic admixture, thereby constraining increases in genetic diversity and subsequent potential gains in fitness or adaptive potential that can promote invasion success (Qiao et al. 2019). Restricting access to or affecting the habitat suitability of sites that hold high centrality within the network of suitable habitats can substantially contribute to this objective. Indeed, limiting movement through these key nodes disproportionately reduces the likelihood of foci merging and thereby slows large‐scale population consolidation (Drake et al. 2017; Tingley et al. 2012). Similar logic may also apply to microscopic invaders with complex dispersal networks.

While the conceptual framework developed here is primarily optimized for macro‐organisms (plants and animals), where invasion fronts are more readily observable and direct management interventions more feasible, analogous core‐to‐front evolutionary and ecological dynamics can also arise in microscopic invaders. For example, the amphibian chytrid fungus (*Batrachochytrium dendrobatidis*) shows evidence of increased virulence at the invasion front relative to established populations during its spread through Central America, consistent with spatial sorting and rapid evolution (Phillips and Puschendorf 2013). In host‐specific pathogens, where the host itself acts as the primary dispersal vector, the same principles of identifying and managing infected versus uninfected individuals or populations can be applied within this framework (Hulme et al. 2020). However, for many fungi, nematodes and other microscopic invaders that rely on passive long‐distance dispersal (e.g., via wind or water) or where environmental suitability dominates over active vectors, tracking and managing invasion fronts present greater practical challenges due to lower detectability and fewer direct intervention options. These cases often benefit from molecular tools such as eDNA for mapping fronts and from broader epidemiological approaches that integrate invasion biology with pathogen dynamics (Hulme et al. 2020), although they may require extensions of the concepts presented here.

### 5.4 Concrete Interventions at the Front

A practical approach could involve using physical or chemical barriers to prevent access to critical stepping‐stone sites or to delay or stop the advance of the front (Jones et al. 2021). This strategy has been widely applied across ecosystems: fences in Australia have been used to contain rabbits, foxes and cats (Dickman 2011), electric barriers limit dispersal of invasive fishes in the USA (Collins et al. 2024), and other structures, such as dams and weirs, can restrain the movement of non‐native species (Carvalho et al. 2025; Jones et al. 2021). Although these strategies are effective in containing certain invasive species, such barriers can have an impact on native communities by fragmenting their habitats (Jones et al. 2021; Bradby et al. 2014). Furthermore, while these barriers prevent certain species from proliferating, they have enabled other invasive species to spread (Brown et al. 2006). These management interventions should not be limited to source populations. From a practical standpoint, sink populations (currently non‐reproductive or limited by environmental constraints) are frequently disregarded because they do not contribute to immediate spread. However, environmental or anthropogenic changes can transform these sinks into viable sources, converting dormant populations into new invasion fronts. For example, introduced trees may not spread until compatible mutualists are also present. In Pinaceae, invasion can be strongly limited by the absence of ectomycorrhizal fungi, with spread often remaining concentrated near plantations where suitable fungal inoculum is available (Nuñez et al. 2009; Spear et al. 2021; Le Roux et al. 2017). Moreover, sites colonized near the range edge may initially function as sink habitats, but as the invasion front advances, they may transition into source sites, contingent on local habitat suitability (i.e., not being intrinsically limited by persistent abiotic constraints such as soil or water chemistry; Everts et al. 2023). Therefore, proactive management must incorporate the potential future activation of sink populations within adaptive surveillance frameworks. Anticipatory treatment of sinks can prevent the sudden emergence of new fronts, especially in systems prone to environmental regime shifts or human‐induced landscape changes. While technically feasible, the application of these management measures in the field can be constrained by different jurisdictions during a cross‐boundary invasion (Everts, van Doorn, et al. 2025; Haubrock, Everts, Kouba, et al. 2026), conflicting management priorities among agencies (Simberloff 2020), no access to privately owned sites (Vimercati et al. 2017), and low detectability and latency of early‐phase populations (Bylemans et al. 2016). While many of the interventions discussed in this section are most relevant to macro‐organisms, certain principles (such as targeting infected hosts or vectors as key sources) have direct parallels in the management of some host‐specific pathogens (Hulme et al. 2020).

## 6 Conclusion and Future Directions

Invasion fronts have been studied for decades through ecological and evolutionary theory and conceptual models, long before high‐resolution movement data became available. Classic work on diffusion‐like spread (Okubo and Levin 2002; Skellam 1951), Allee‐effect dynamics (Kot et al. 1996; Lewis and Kareiva 1993), and patchy or fragmented spread frameworks (Andow et al. 1990; Hengeveld 1989) laid the foundation for our understanding of how fronts form and propagate. Recent technological advances now complement this theoretical framework, with increasingly detailed organism‐tracking data providing new insights into processes at invasion fronts. Although researchers are applying sophisticated modelling approaches to these expanding datasets (e.g., artificial intelligence and machine learning; Zeng et al. 2021; Schlägel et al. 2019; Elith et al. 2008), identifying the cues and mechanisms that drive invasion fronts remains challenging. Specifically, the increasing availability and resolution of molecular tools allow a better understanding of the gene‐level drivers and consequences of range‐expansion (Kołodziejczyk et al. 2025). Modern tracking technologies now deliver not only high‐resolution movement data but also information from accelerometers and physiological sensors, producing comprehensive, individual‐level data streams for non‐native species (Meira et al. 2024). These tools allow researchers to provide deeper insight into the factors that shape core‐front invasion dynamics, and—building on the integrative framework and front typologies synthesized here—when integrated with ecological context and landscape structure, enable a shift from pattern description to mechanistic prediction of when, where, and how invasion fronts emerge, accelerate, stall or collapse. However, missing links that limit our ability to recognize invasion fronts effectively remain. These include sparse data on low‐density front populations, limited temporal resolution for detecting rapid front shifts, and the lack of models that integrate occurrence, movement, genetic and environmental data under non‐equilibrium conditions. Additional constraints arise from imperfect detectability, especially for cryptic or microscopic invaders and low‐density populations, and from limited translation of monitoring outputs into operational management. Thus, the main bottlenecks are not only data availability, but also data integration, mechanistic modelling and management implementation. Together, this synthesis reframes invasion fronts from descriptive spatial patterns into dynamic eco‐evolutionary systems. Beyond advancing fundamental ecological and evolutionary insights, core‐front invasion dynamics can also have substantial implications for the design and implementation of invasion management strategies.

## Author Contributions

P.J.H.: conceptualization, writing – original draft, writing – review and editing, visualization. N.A.A.: conceptualization, writing – original draft, writing – review and editing. S.K.: writing – original draft, writing – review and editing. F.E.: writing – original draft, writing – review and editing. A.S.T.: writing – original draft, writing – review and editing. J.P.: writing – original draft, writing – review and editing. P.E.H.: writing – original draft, writing – review and editing. D.A.A.: writing – original draft, writing – review and editing. A.B.N.: writing – original draft, writing – review and editing. T.E.: writing – original draft, writing – review and editing. C.H.: writing – original draft, writing – review and editing. R.S.: writing – original draft, writing – review and editing. J.R.B.: conceptualization, writing – original draft, writing – review and editing, supervision.

## Funding

This work was supported by the European Commission under the European Union’s Horizon Europe research and innovation programme, Marie Skłodowska‐Curie Postdoctoral Fellowship grant agreement No. 101203662 and grant agreement No. 101181413; by the Portuguese Foundation for Science and Technology (FCT) through COMPETE2030‐FEDER‐00686700; and by the Austrian Science Fund (FWF), grant I 5825‐B. Funded by the European Union. Views and opinions expressed are however those of the author(s) only and do not necessarily reflect those of the European Union or the granting authority. Neither the European Union nor the granting authority can be held responsible for them.

## Data Availability

The authors have nothing to report.
